# An Essay on Mechanical Dentistry

**Published:** 1880-08

**Authors:** W. W. Ford

**Affiliations:** Dentist, Macon, Georgia, Chairman of the Committee on Mechanical Dentistry.


THE
AMERICAN JOURNAL
OF
DENTAL SCIENCE.

Vol. XIV. THIRD SERIES—AUGUST, 1880. No. 4.

### ARTICLE I.

### *An Essay on Mechanical Dentistry.*

BY W. W. FORD, DENTIST, MACON, GEORGIA, CHAIRMAN OF THE COMMITTEE ON MECHANICAL DENTISTRY.

(Read before the Georgia State Dental Society. Session of 1880.)

Professor Harris says: "Mechanical Dentistry means the art of constructing and applying artificial teeth, artificial palates, and appliances for the correction of irregularity in the arrangement of the natural teeth." I think Dr. Harris' definition of Mechanical Dentistry is too limited. Mechanical Dentistry, or dentistry that is strictly mechanical, forms a very important part of nearly every operation belonging to the dental profession; this is true also of the profession of general surgery. The extracting of a tooth is mechanical; the knowing how and when to do it is not. The making of a gold or other filling is mechanical; the knowing how and when to do it is not. The making and adjusting of a full or partial set of teeth is mechanical; the knowing how and when to do it is not. So in surgery, the setting and splinting of a broken limb is mechanical; the knowing when and how to do it is not; and even further, the amputating of a limb, the tying of an artery, is after all strictly mechanical, the knowledge of how and when to do it is not. Then I say away with your line of distinction drawn by the word mechanical between the different operations in our profession.

I am firmly of the opinion that operations that are strictly mechanical in our profession are a vast deal more important than many things we write and read and think more about. What does it benefit a dentist or his patrons for him to be thoroughly versed and highly educated in his head, if his hand is not correspondingly educated to put into practice what his head knows? I have known some highly educated dentists, so far as the theories of dentistry was concerned, that could not make a decent filling or perform any mechanical operation in dentistry in even a passable manner. I will, however, say no more on this line of thought, as I fear I am trespassing upon ground that I am not expected to occupy. I will therefore confine myself to that department which is generally considered legitimate—*Mechanical Dentistry.*

I wish I could say something; I wish I could devise some means or construct some argument that could even assist in elevating the standard of this much abused and degraded part of our profession to that high standard where it properly belongs; I wish I could speak in such thundering tones that every dentist in this broad land and even in other lands, might hear me, that I might tell them the great importance of elevating the standard of mechanical dentistry to a higher standard of mechanical education, a higher standard of work, a higher knowledge, a more skillful and artistic application of the rule and laws of artificial dentistry.

But what shall I say to you, dentists of Georgia? What can I say? Will you let me beg you and pray you to stop and think, and look and gaze with me, gentlemen, at the present degraded condition of this useful, scientific and all important part of our honored, noble profession. Shades of the departed spirits of Bell, of Fox, Bourdet, Fouchard,

Kœcker, Delabarre, of Harris, and Robinson, rise up from your mouldering clay, and stand up here to-day in all your immortal glory, and behold with us of the Georgia Association this magnificent ruin of all the faithful efforts that you spent so much time, toil and talent in constructing, building and beautifying.

"Tell it not in Gath, publish it not on the streets in Askalon," that even in this great and glorious Empire State, noted ever for her wealth, her education, her refinement, her intelligence, her liberality and patronage to the arts and sciences, even in the very heart of this State, sets of things, claiming to be sets of first-rate artificial teeth, are made and palmed off upon a credulous patient or patron, with any number of guarantees that they may ask for, for the mighty, the stupenduous sum of five or ten dollars a set, including the extracting of the natural teeth. What a magnificent fee even the latter is for a scientific, skillfully performed operation by an educated, high-toned, proud, professional gentleman.

What a liberal, philanthropic, public-spirited profession we are getting to be. Look at it, gentlemen, and think of a man giving his time and studying hard from three to five years, (for I hold no man can learn it in less, in or out of college,) paying for his own board and clothes, his tuition, buying his own text books, instruments and materials to learn a profession, and when he has done and paid all this, he sets up to practice, and gives away, yes, gives his patrons the entire benefit of all his toil, time and expense, "*free gratis for nothing.*" How grand, how generous, how magnanimous he is. Let his name be written and indelibly engraved in letters of living light athwart the ethereal blue, that it may last and rise up to the full duration and dignity of immorality. I am led to exclaim, in the beautiful language of Poeck, "may his lovely and beautiful shadow never grow less or refuse to follow him whithersoever he goeth."

I say, and take it for granted, that all his knowledge, all his skill, all his grand and magnificent talents and time are given away when he works at such a price, because my material, board and clothes, without putting in office rent, and taxes, and license, costs me fully that much to make a set of teeth that I would have the hardihood and audacity to put in my patient's mouth and tell him or her they were worth carrying home, or wearing out of my office.

> "Oh wad some power the giftie gie us,
> To see oursels as ithers see us."

Let us diagnose this case and see if we can find out the cause of this flood of cheap stuff that is scattered all over the country. I am of the opinion that it is in a great measure the result of poor, cheap material that has been thrown upon the market, together with the plastic materials used for plates, and also the want of mechanical skill. In the old and better styles of plate work, on gold and platina plates, no man could get off a piece of plate work and get the money for it unless he was able to do a fair job, because he could not make it stick in the mouth long enough to get it off his hands. It required more skill, more mechanical education, if you please, both of the head and hand, to make it do at all, and much more than these plastic materials do. With them, any man that can take an impression and make a plaster model—can, in a few hours, get up something that will, after wearing for a few days, answer for awhile. I knew one dentist who told his patient if the plate did not fit well, to soak it in a cup of hot coffee and put in the mouth while warm and press it up and it would be a.l right. The great trouble about this work is, the people's natural teeth are being sacrificed by the wholesale to give place to these miserable abortions known as cheap sets of teeth, which is recommended as being better than the natural ones, because they will not ache. Oh how anxious everybody is to get rid of the tooth-ache once for all; how ready they are to believe anything in that direction and suffer anything else but that. Well, these teeth they get may never ache, but they make many hearts ache when they realize the loss of their natural teeth when it is too late.

When I first entered the profession, twenty-five years ago, things were not so. The cheap men of those days did not pull out teeth as they do now, because they could not put them back, consequently they would fill them with something, either gold, or tin foil, or common silver amalgam, or Hill's stopping, or something that did no harm, if it did no good. They at least left the teeth in the mouth so the person had another chance of having them saved by somebody that could do it. Now, it is easier to pull them out and put in a worthless something in their place than it is to even try to save them.

But we will not pursue this thought further. We will now give our views, in part, at least, as near as we can, of what we consider some of the requirements and duties of the mechanical dentist in his laboratory.

I hold, that no man can learn to be an accomplished mechanical dentist without, what I would term, a regular apprenticeship under a competent and accomplished preceptor in the laboratory. The principal trouble of the present day is, that we learn, or think we learn, it too soon. We do not spend the time and study absolutely necessary to learn even the fundamental principles of it, much less the art and training of the hand and eye so necessary to a fine execution of the work, and then we are too easily satisfied ourselves. If the patient does not complain it is all right with us, whether we may know ourselves that it is not, or whether we may know that it is, all the same with us. The amount of time required to obtain the knowledge and practice this part of our profession successfully, will of course vary with different men, owing entirely to their mechanical genius and aptness, and also to their former education in the arts and sciences. Some men are so quick to gather and put in practice any mechanical or scientific idea, that it does not take them long to become an expert in mechanism of any kind, while another may dig and dig for a lifetime and then be ordinary, or low middling; but a certain amount of knowledge and practical ability is absolutely necessary to success with all, and it takes some time for all to obtain this knowledge and ability, and not only time, but a competent preceptor or teacher, for if a man learns wrong it is much harder for him to get right than it is to start from the beginning. No man can just look at two or three good pieces of dental mechanism and learn from that, or see a few pieces made by another and learn from that. He must learn to do it himself, and do it himself unaided, because the first case he has may require something entirely different from anything he ever saw. The accomplished, successful mechanical dentist must be to all intents and purposes an artist; he must study the human face as well as the laws of nature there developed; when natural teeth are gone, as is often the case, before he ever sees the face, he must be able to look at that face and see and know exactly what that face has lost and what is needed to restore it to its orignal beauty, symmetry and regularity. The expert will not go to work and try to improve upon nature in perfection by making what some would call a beautiful, small, white, pearly set of teeth for a large, dark back ground. The true artist copies nature as closely as possible. If the scene selected is a rough, scraggy mountain, or a boiling, tempest-riven sea, in painting or transferring the scene to canvas, he sticks to the natural original as it is, and paints it as he sees it in his mind's eye. He would not paint a beautiful flower garden on a sandy, storm-beaten beach, or one on the scraggy, snow-capped summit of a high mountain. He will paint them on the lawn or the meadow, where the flowers usually grow. On the scraggy top of the highest mountain he will paint the beautiful snow. He will make things have their natural fitness and correct adaptation. Thus it is with the artistic dentist; he will study nature closely and diligently in all its different phases as depicted in the human face and copy after it, and not assume the prerogative of the "great Creator" by trying and claiming that he can improve upon it. In dentistry, true art is in concealing art, by imitating nature so closely and perfectly that the difference will not be discovered.

The next thing, he must thoroughly understand the nature and the composition of the material that he is working with. I know there are many men trying to work porcelain teeth, that are totally ignorant of what and how they are made; there are some who use rubber and vulcanize it, that know nothing ot the composition of dental rubber (or caoutchouc) and cannot tell why it is that a certain amount of heat hardens it. A dentist told one of my patients this year, that he would not make or wear a rubber plate because the rubber had arsenic in it. I knew that all of the dental rubbers had the sulphuret of mercury known commonly as vermilion, in them as a coloring matter, but that was the first time I had heard of arsenic being in it. Now no man can be a success in medical dentistry unless he thoroughly understands all about what he is working with and upon, the whys and wherefores; it cannot be made a success in the dark. He must know all about what he is doing in every part of the entire process, and next what is not of the least importance, he must be a high graduate, fine, close seeing, tasty, skillful, ingenius, well trained mechanic. If the dental profession were all better mechanics than they are, both in the operating room and in the laboratory, we, and the world would be better off than we are now. I claim that it takes a high order of mechanical skill to produce a first rate piece of artificial dentistry, on any kind of a plate, whether it be gold, platinum, rubber or celluloid; whenever you look at the work, you can tell very quick, what sort of a mechanic the man was that made it, however more difficult one process may be than another, still you can detect in either, what manner of man has been at work on it.

A first class mechanical dentist is able to make a good job of work on any base that is now made or worn. No man is fully competent to practice this department of our profession who is only competent to make rubber or celluloid work. Every dentist that sincerely desires to excel and reach the highest point of excellence in this department, and cares for the prosperity and elevation of this part of the profession, should learn not only the mechanical, but also the ceramic department, so that he will know how to select the crude minerals and metals from nature's laboratory, and prepare and compound them and form them into those beautiful imitations of the natural organs we see displayed in porcelain teeth, then when he goes to select a set of teeth for his patient, it makes no difference with him who made them, he can readily tell whether or not they possess the requisite strength, the proper blending of colors in the enamels, the proper amount of translucency or not to sustain the proper shade in the mouth, and whether they have been fused enough or too much; this knowledge is almost indispensable with every intelligent dentist, and the only way I know for him to learn it thoroughly, is for him to learn how to make porcelain teeth himself from the crude minerals and metals as they are found in a natural state; this part of our profession, for I claim that it is a legitimate part of it, is by far the most beautiful, interesting, and scientific. I could never tire of those beautiful and careful operations and processes by which porcelain teeth are produced; the knowledge is worth the time and labor it takes to learn it as a matter of pleasure and information, if a man never had any use for it. But every dental mechanician has almost daily use for this knowledge; he should not be found without it.

Every mechanical dentist ought to thoroughly understand the nature and character of nearly all the different metals, particularly the royal metals. He should understand how to melt, weld, temper, refine, solder or alloy them to any degree of fineness, so he will know how to adapt them to his case that he may need them for, and how to make or alloy his solder, what grade of fineness to have it for the piece of plate or wire he wishes to unite by fusion, and what kind of flux to use for the different metals to make them work to the best advantage. In gold or continuous gum work on platina plate, he needs a good knowledge of moulding in sand, so as to transfer any plaster model that he may have to a correct duplicate in metal.

The next point I will call your attention to is, the mechanical dentist in all work and in all cases must be a master with the laboratory lathe and corrundum wheels. If working on gold, he must fit his tooth or teeth nicely down on the plate at every point, so it will bear equal at every point; he must make his joint square against the adjoining teeth, to fit as close inside as outside, and at the same time have the proper length and contour. In rubber or celluloid, if he is working gum teeth, (and no others are so good in nearly all cases for either of these bases,) he will, in order to keep the material of the plate out of his joints, which destroys the beautiful natural effect of a good porcelain gum, he will be exceedingly careful to make his joints fit very close on the inside as well as the outside, for if the rubber once starts into a joint it will go through and discolor it; one brother said to me, "I can't keep the rubber out of my joints any way I can fix it." I will tell you how to do it—in the first place, you must have a good, true, steady running lathe. I found Snowden & Cowman's United States lathe a first-rate machine. I like the short spindle best; I am now using S. S. White's laboratory lathe, No. 3, and I am very much pleased with it; those who wish to sit down to the lathe had better get No. 4. Place your lathe near a window or under a good sky-light, get a corrundum wheel that runs perfectly true on your lathe, (I use Johnson & Lund's, with a brass centre with threads cut in them,) see that the wheel is also trne on the face; if you can't see a chigger's eye, get you a good pair of spectacles, hold your section or tooth square and steady against the face of the wheel, and then if you can't make a good joint you are a poor mechanic. If the face of your wheel is worn out, as I call it, full of ridges or furrows, you cannot make a close joint with it—cast it aside and get a new one. My friend and colleague, Dr. W. R. Holmes, told me he used a new wheel for every set of teeth he made. I have never found it necessary to use that many, but I discard a wheel just as soon as it gets so uneven on the face that I cannot grind my section perfectly true and square with it. Be careful not to grind away too much of the material before you try it to its place, then you can tell where it wants grinding off the most before your material is gone. The block or section should never be held to the wheel so it will cut from the inside towards the outside of the gum, because it will flake off or notch up the edge of the gum enamel, which is softer than the body, and will give you a rough, ugly joint just where you want it smooth and even ; next, after making your joint as close as you can, and you find after removing your base plate, that your joint is the least bit open on the inside, fill it up with dry plaster, and wet it with liquid silex, that will keep the plate material from starting in it; if you find your joint is very open, better let the rubber go in and fill it up, as it is better filled up with rubber than left open to catch food and secretions of the mouth. I measure my rubber in a star rubber gauge, being careful to get as near as possible the exact amount needed ; after packing, I bring my flasks together slowly in boiling water. I use an old oil celluloid boiler and press for that purpose. After it is brought entirely together, I take it out and put in my bolts. I think dry heat should never be used with rubber ; it destroys all the vitality and life of it, and makes it weaker. I am careful not to have too much excess of rubber, if you do in many cases it will press the teeth back and open your joints and rubber them, however close you may have made them. I cut notches with a file on the bottom edge of the upper section of my flask and cut my gates to those notches, so if I get too much excess of rubber it will squeeze entirely out of the flask, and not spread between the sections of the flask and hold them apart or resist the pressure so stubbornly as to move the teeth before it will get away.

I will call your attention to another fact just here. If the teeth are tried in the mouth on the base plate after they are ground up and the saliva gets in the joints, they ought to be taken off the base plate and all the saliva washed off them thoroughly with warm water and the bicarbonate of soda; if they are cased up in the flask with saliva in the joints, when they are vulcanized (or soldered, if on gold,) the joints will turn perfectly black, however good otherwise they may be, and you cannot possibly get the black out and get them bright again; even nitric or hydrochloric acid or alcohol will have no effect upon them. On this point of good and bad joints I have tried to be a little explicit, even at the risk of being considered tedious, from the fact that I have seen more universal failure just here than any where else. Still I am saying nothing in this essay, I presume, that will be new to many of you, neither do I hope to do so, but it may be of interest to some to listen to the recital of these important truths.

### REPAIRING RUBBER PLATES.

There is only one way to repair a rubber plate when it is cracked or broken entirely in two pieces that will make it good again, and that is to duplicate the plate, and make the rubber part of the job entirely new. This process was described by Dr. W. R. Holmes, in No. 2, of the *Dental Luminary*, some time ago. I will describe the way I do it as near as possible. Some ten or eleven years ago I had trouble with some plates that split or got broken. I tried the old way of boring holes through the plate and putting on a patch of new rubber. I found the new rubber did not unite with the old, neither did the crack in the old plate unite together—the only strength given to the job was the strength contained in the patch, and by vulcanizing the old plate over it was damaged and very much weakened. The thought occurred to me that I could cast a model and counter model of the old plate, and by the aid of heat pull out all the old rubber and then obtain a perfect duplicate without moving or loosing the teeth, or my articulation.

I succeeded so well, that now I never repair a broken plate any other way. If the plate is broken entirely in two, stick it together with liquid silex, then drop some melted wax on the crack on the lingual surface of the plate, then fill the bottom of your flask with soft plaster and after oiling the surface, fill the palatine surface of the plate and press it down upon the plaster in the flask until the plate is thoroughly full and comes over the edge up to the edge of the porcelain gum of the tooth. After trimming as in a new job, fill up the other section of the flask, after scraping away the wax you dropped on the crack in the old plate. When the plaster has thoroughly set, take the flask carefully apart; you will find the edges of the plate exposed so you can take hold of it with nippers or plyers, then put the section containing the teeth and plate in water and boil it strong for ten minutes, take it out and take hold of the edge of the old plate with nippers quickly and pull it all out. If hot enough it will pull out easy and perfectly. If you want the new plate thicker, scrape away the plaster of the counter-model containing the teeth, cut gates and proceed by packing, etc., as in a new job; after doing this a few times you will find you can do it nearly as quick and as easily as you can patch a job. You will then have a new plate that is obliged to fit if the old one did, for you have a perfect duplicate in every particular, with no old revulcanized burnt rubber to trouble you or your patient again.

The arrangement of the teeth on the plate is a part of the work that is done wrong as often, or more often than it is done right. I have noticed that a great many in grinding up and articulating a set of teeth just let them go any way they will without any reference to the shape or contour of the face whatever. The result is, we see sets of teeth that when the patient laughs you can see all of the fourteen teeth, and if there was a gold filling put in the posterior surface of the second molar, you could see that. Mouths treated that way when the person laughs, not only look like they had fourteen teeth, but they look as if they had at least forty; the whole face is teeth, you can't see anything but teeth. That is an unpardonable mistake. Don't let your teeth just go into any shape the alveolar border may incline them to go, but make them go as you want them and as the face requires they should go. If the face be narrow, make the teeth narrow, whether the gums are or not, and see that the cuspids nearly cover the bicuspids and the bicuspids cover the molars, and let them run in a straight line from the cuspids back, inclining a little out. If you have a broad face, make them square, or nearly so, in front, and wide between the cuspids and then run nearly straight back on a line behind the cuspids. In other words, from the angle or turn in your teeth on the cuspid teeth and don't make them in a perfect circle, or in the shape of a mule shoe, and never make pointed or scooter-shaped set of teeth for a broad, round face, if you do you will disguise your patient. Study and carefully observe the natural teeth in different shaped mouths and faces; you will see how they are arranged there and copy after them and you cannot materially err.

I would like to have said more on the subject of gold work, but time and your patience forbid. This paper is already extended to too great a length. The whole subject of mechanical dentistry is too much for one paper, or one committee; they cannot do the whole subject justice in the time allotted to them. It ought to be subdivided as the other parts of our profession are and given to more than one committee, but as there is so little gold work and continuous gum work being made in the bounds of our society, I thought if I left out any thing I had better leave out that. I think, and firmly believe, however, that the very best work that can be made, at anything like what is considered a reasonable price in this day and generation, is a good gold plate with sectional gum teeth (made for rubber work,) fastened on it with hard rubber. When well made, it is as strong as all gold work, and stronger than all rubber and as clean and nice as continuous gum work; it does away with all low grade solder that is liable to oxidize in the mouth; it is light and pleasant in the mouth; it can be repaired, if the teeth are broken, with more facility than any other kind of work. I have a piece of that kind of work I made ten years ago, and will show it to any one that may wish to see it. When made, as that is made, it does away with all the objections to either gold or rubber work. I think, however, that Dr. John Allen's continuous gum work, made as he makes it, is the best work ever made without any exception, but it is entirely too costly for the South.

I am not opposed to rubber or celluloid, *per se.* I think they are good in their place, but still I think they have done dentistry a great deal of harm, and have done the public a great deal of harm and a great deal of good. I do not use celluloid, because it never gave me satisfaction. I have never yet seen a piece of it that had been worn over a year but what it had absorbed the secretions of the mouth and became offensive, and had changed its color and in time it loses its strength and toughness and becomes hard and brittle. It may answer a valuable purpose for temporary teeth. Good rubber well and carefully vulcanized and worked with good gum teeth, makes a very good, cheap set of teeth. Two or three sets ought to last an ordinary lifetime, and will cost less at a good price for it than one set on gold or continuous gum work, but like every kind of work, it requires to be well made. A few words more and I am done.

Mechanical dentistry is not receiving the attention of the profession at large that its merits and importance demands. Many of our (so-called) scientific men are disposed to throw it off and discard it. It is not written about in our journals, and talked about in our associations and conventions as much as it ought to be; men that cannot make a first rate set of teeth even on a rubber base, would rather hear themselves gas on the microscopy of a leaf or a fly's foot, or talk about cutting one eighth of an inch off the bottom end of the pulps of a tooth and saving it alive permanently, than to write and talk about mechanical dentistry, because these terribly scientific gentlemen are afraid they will be classed as mechanical men, when the truth is, if their true mechanical ability was known to the world they would not be in the least danger of being classed among good mechanics. I am not opposed however, to a proper study of the microscope or the conservative treatment of the dental pulp, but let us first learn how to do in the best manner possible all the legitimate and every day operations of our profession, then we will have time to study those subjects more thoroughly. Prof. Charles Essig said one dental student wanted to matriculate in the dental department of the University of Pennsylvania, just to learn operative dentistry; he just wanted to learn how to ply teeth; he did not wish to know or learn any thing about what he called mechanical dentistry; of course he was not admitted on those terms, and never could be, where as fine a mechanical dentist as Prof. Charles Essig is, was at the helm. Whenever you see a man turning up his nose at this department, and sending off his plates to somebody else to make, you may just set it down as dead certain, that he cannot make a good job to save his life. First class men in this line, are not willing to trust their work in any one's hands but their own, especially in the hands of some one who never saw their patient's face or mouth. They will take special pleasure and delight in doing it themselves. In conclusion, we will say there is not a word in this paper that is meant or intended to be personal to any dentist in Georgia. Now let us one an all, dentists of Georgia, strive harder than we have ever done before in every job that we make, not only in the laboratory, but also in the operating room. To reach the highest point of excellence, let us place upon our banners the grand old motto of Homer: "*Strive for the Best*," until we all get as near perfection as it is possible for mortal, finite man to reach.—*Dental Luminary.*

